# Functional Response and Predation Potential of *Arma custos* (Fabricius) Against *Tuta absoluta* (Meyrick)

**DOI:** 10.3390/insects17101060

**Published:** 2026-10-14

**Authors:** Yuyang Jiang, Yan Zhao, Xiujie Li, Linli Peng, Sheng-Yen Wu, Youming Hou

**Affiliations:** State Key Laboratory of Agricultural and Forestry Biosecurity, Fujian Agriculture and Forestry University, Fuzhou 350002, China; 15290237632@163.com (Y.J.); zhaoyan1996@126.com (Y.Z.); 19861909770@163.com (X.L.); 15736654542@163.com (L.P.)

**Keywords:** the predatory stink bug, the tomato leafminer, predatory functional traits, intraspecific interference, greenhouse cage trial, olfactory preference

## Abstract

The tomato leafminer, *Tuta absoluta* (Meyrick) (Lepidoptera: Gelechiidae), is a globally invasive pest causing severe economic losses in tomato production. We evaluated the predatory stink bug *Arma custos* (Fabricius) (Hemiptera: Pentatomidae) using functional-response assays, a predator-density experiment, behavioral observations, Y-tube olfactometer bioassays, and a greenhouse cage trial. Depletion-aware maximum-likelihood models supported a Type III response for adult females feeding on second-instar larvae, whereas Type II models were retained for fifth-instar nymphs and adults feeding on fourth-instar larvae. At an initial density of 40 larvae, females consumed 16.2 ± 0.2 second-instar or 17.4 ± 0.5 fourth-instar larvae within 24 h. With 40 fourth-instar larvae available per arena, increasing female density from one to five reduced per capita consumption from 17.40 ± 0.51 to 5.44 ± 0.04 larvae per day. The Hassell–Varley model provided the best fit (*m* = 0.725; 95% CI, 0.693–0.755). In greenhouse cages, releasing three females per cage reduced cumulative damaged leaves by 60.12% by day 15. No active larvae were detected in release cages at that time, compared with 11.33 ± 0.33 in controls. Females preferred infested over healthy tomato plants, whereas males showed no significant preference. These findings demonstrate that *A. custos* is a promising biological control agent for integrated pest management (IPM) of *T. absoluta*.

## Simple Summary

The tomato leafminer *Tuta absoluta* (Meyrick) (Lepidoptera: Gelechiidae) is a destructive pest whose larvae feed concealed within tomato leaf mines and are therefore difficult to control. We evaluated the predatory stink bug *Arma custos* (Fabricius) (Hemiptera: Pentatomidae) using laboratory feeding and predator-density assays, behavioral observations, olfactometer bioassays, and a greenhouse cage trial. Depletion-aware models supported a Type III response by adult females to second-instar larvae, whereas Type II responses were retained for fifth-instar nymphs and adults feeding on fourth-instar larvae. Adult females also showed consistently high consumption across the larval stages tested. Per capita consumption declined as predator density increased in small arenas. In greenhouse cages, predator release reduced detectable active larvae and cumulative leaf damage, while adult females oriented toward infested tomato plants. These findings support further greenhouse and field evaluation of *A. custos* as a complementary natural enemy, not a demonstrated replacement for insecticides.

## 1. Introduction

The tomato leafminer, *Tuta absoluta* (Meyrick) (Lepidoptera: Gelechiidae), is one of the most destructive invasive pests of tomato worldwide. Since spreading beyond its native range in South America, it has become established across major tomato-producing regions in Europe, Africa, and Asia, where its larvae damage leaves, stems, and fruits, and can cause substantial yield losses under severe infestations [1,2,3,4]. Management of *T. absoluta* continues to rely heavily on synthetic insecticides [5,6,7]. However, its concealed leaf-mining habit, short generation time, overlapping generations, and rapid evolution of resistance frequently compromise chemical control [1,2,3]. Field populations have also rapidly evolved resistance to multiple insecticide classes [5,6,7]. These constraints create an urgent need for biologically based tactics that can be incorporated into integrated pest management programs.

Predatory hemipterans are important natural enemies of *T. absoluta* because they can actively search for prey and suppress pest populations before extensive crop damage occurs. Mirid predators such as *Nesidiocoris tenuis* (Reuter) and *Macrolophus pygmaeus* (Rambur), together with anthocorids and coccinellids, have shown considerable potential against the pest [8,9,10,11,12]. Their effectiveness, however, is often influenced by prey developmental stage and accessibility, with many species performing most effectively against eggs and young larvae [8,9,13,14,15]. Larvae that survive early-stage predation may therefore remain insufficiently controlled as they grow and continue feeding within tomato tissues. This stage-dependent limitation highlights the need to identify complementary predators capable of suppressing larval stages of *T. absoluta*.

*Arma custos* (Fabricius) (Hemiptera: Pentatomidae) is a predatory pentatomid that attacks a broad range of lepidopteran larvae, including several economically important agricultural and forestry pests [16,17,18,19,20,21]. Its relatively large body size, ability to subdue mobile caterpillars, and established artificial-rearing methods make it a plausible candidate for augmentative biological control [21]. Prey acceptance or high consumption under simplified laboratory conditions, however, is not sufficient to establish the practical value of a predator. Effective biological control depends on several interconnected ecological processes: how prey removal changes as pest density increases, whether competition among conspecific predators constrains the efficiency of mass release, and whether the predator can locate prey-infested plants through plant-mediated chemical cues [22]. Functional response, intraspecific interference, and olfactory orientation therefore provide complementary information for determining whether laboratory predatory capacity can translate into crop protection under more realistic conditions. Previous studies have evaluated *A. custos* against *T. absoluta* in combination with *Beauveria* fungi or entomopathogenic nematodes [23,24]. These studies addressed combined-agent effects rather than jointly evaluating prey-density responses, predator-density effects, olfactory orientation, and plant protection. The present study extends this evidence by linking these processes in a coordinated laboratory and greenhouse cage assessment, addressing whether larval predation by *A. custos* translates into reduced tomato leaf damage.

We consequently tested three related hypotheses. First, functional-response form or fitted parameters would vary among fifth-instar nymphs, adult females, and adult males of *A. custos*. We also expected differences between second- and fourth-instar prey tested with adult females. Second, increasing predator density under fixed total prey availability would reduce per capita consumption. Third, olfactory orientation would vary by sex and odor source, while predator release would reduce detectable larvae and cumulative leaf damage in greenhouse cages. We combined functional-response assays, a predator-density experiment, behavioral observations, Y-tube olfactometer bioassays, and greenhouse cage trials to evaluate these predictions.

## 2. Materials and Methods

### 2.1. Plant Cultivation and Insect Rearing

Tomato plants, *Solanum lycopersicum* L. cv. Zhefen 202, ranging from 24 to 31 cm in height and bearing six to eight fully expanded leaves, were used for both insect rearing and all laboratory and greenhouse cage experiments. Seeds were sown in plastic pots (9.5 × 4.5 × 8.5 cm) filled with commercial potting substrate. Plants were watered every two days, fertilized once weekly with nitrogen fertilizer, and maintained without pesticide application in a climate-controlled chamber at 25 ± 1 °C, 60 ± 5% relative humidity (RH), and a photoperiod of 16L:8D.

A laboratory colony of *T. absoluta* was established from larvae collected in Yuanmou, Yunnan Province, China, in May 2024. The *T. absoluta* colony had been maintained on tomato plants for 10 laboratory generations before the experiments. Insects were reared on tomato plants in mesh cages (50 × 50 × 50 cm) under the environmental conditions described above.

A colony of *A. custos* was obtained from Jiyuan Baiyun Industrial Co., Ltd., Henan Province, China, in July 2024. Following receipt from the supplier, the *A. custos* colony had been maintained in our laboratory for eight generations before the experiments. The insects were maintained in ventilated plastic containers (20 × 13 × 8 cm), with ventilation openings covered by fine mesh. First-instar nymphs were supplied with a 10% honey solution, whereas later-instar nymphs and adults were fed pupae of *Antheraea pernyi*. Folded green paper strips were provided as oviposition substrates.

### 2.2. Predatory Performance of Different Predator Stages, Sexes, and Prey Instars

We tested fifth-instar nymphs (24 h old) and adult females and males (7 d old), both unmated, against fourth-instar *T. absoluta* larvae (24 h old). Adult females were also tested against second-instar larvae (24 h old). Assays were conducted in ventilated plastic containers (7.2 × 5.5 × 3.5 cm). Before each assay, predators were deprived of prey for 24 h and supplied with water on moistened cotton. Ten tomato leaflets and 5, 10, 15, 20, 30, or 40 *T. absoluta* larvae were placed in each container, after which one predator was introduced. Each combination of predator category and prey density was replicated five times. Predator-free controls were established at each prey density to quantify natural larval mortality. All containers were maintained under the controlled environmental conditions described in Section 2.1. After 24 h, the number of surviving larvae was recorded, and the number of consumed larvae was calculated from the difference between the initial and surviving prey numbers. At the end of each assay, larvae were individually examined. Larvae were classified as consumed when they had lost mobility, showed body collapse, desiccation, or rigidity, and did not respond to gentle stimulation with a fine brush. Intact and responsive larvae were recorded as survivors. Prey were not replaced during the assay.

Because prey were not replaced and a substantial proportion was consumed, depletion-aware models were fitted in R v.4.5.2 using the frair package v. 0.5.203 [25], and parameters were estimated by binomial maximum likelihood:

Rogers random-predator equation (Rogers II; Type II) [26]:

(1) $$N_{e}=N_{0}-\frac{W\left(a\cdot h\cdot N_{0}\cdot \exp\left(-a\cdot \left(T-hN_{0}\right)\right)\right)}{a\cdot h}$$

where *N_e_* is the number of prey consumed, *N*_0_ is the initial prey density, *a* is the attack rate, *h* is the handling time (d), *T* is the experimental duration (1 d), and *W* denotes the Lambert-W function. The flexible power-law extension of the Rogers model (flexpnr) is expressed as follows [25,27]:

(2) $$N_{e}=N_{0}-\frac{W\left(b\cdot {N_{0}}^{q}\cdot h\cdot N_{0}\cdot \exp\left(-b\cdot {N_{0}}^{q}\cdot \left(T-h\cdot N_{0}\right)\right)\right)}{b\cdot {N_{0}}^{q}\cdot h}$$

In the flexible power-law model, the attack rate is expressed as *a* = *b*·*N*_0_*^q^*, where *b* is the search coefficient and *q* is the scaling exponent. Setting *q* = 0 reduces the model to the Type II form, whereas *q* > 0 produces a Type III response.

Parameters were estimated by maximum likelihood with a binomial error distribution using frair_fit [25,28]. The Rogers equation was evaluated with the Lambert-W function. Models were compared using AICc [29]. Under a prespecified parsimony rule, flexpnr was retained only when its AICc was at least 2 units below that of Rogers II. Parameter uncertainty and fitted-curve confidence envelopes were obtained from 999 nonparametric bootstrap resamples of independent arenas within each initial prey density using frair_boot. Because the attack coefficients a and b have different meanings in the two response forms, they were not compared directly across model types. Model-derived 1/h values were treated as descriptive transformations rather than formal rankings.

### 2.3. Interference Assay and Model Fitting for Among Adult Females

To test whether increasing predator density reduced individual predatory performance, adult-female *A. custos* were evaluated at densities of 1, 2, 3, 4, or 5 predators per container. Before the assay, females were deprived of prey for 24 h under the conditions described above. Forty fourth-instar *T. absoluta* larvae and ten tomato leaflets were introduced into each experimental container, followed by the designated number of predators. Each predator-density treatment was replicated five times. After 24 h, the number of surviving prey was recorded, and total and per capita prey consumption were calculated.

We quantified the effect of predator density on per capita consumption using two classical interference models fitted by maximum likelihood. Both were compared with *a* null model of no interference. Each model expressed per capita consumption *E* (prey predator^−1^ d^−1^) as *a* function of predator density *P*. Initial prey density remained fixed at 40 larvae per arena.

The Hassell–Varley interference model (power-law form) [30]:

(3) $$E=Q\cdot P^{-m}$$

Here, *Q* is the quest constant, or per capita consumption at *P* = 1. The mutual interference coefficient *m* quantifies the density-dependent decline in per capita feeding, with *m* > 0 indicating interference.

The Beddington–DeAngelis interference model (simplified for fixed prey density) [31,32]:

(4) $$E=\frac{E_{0}}{\left[1+\gamma \cdot \left(P-1\right)\right]}$$

Here, *E*_0_ is per capita consumption at *P* = 1. The interference coefficient *γ* describes the decline in feeding associated with encounters among predators. We also fitted a null model in which per capita consumption was independent of predator density (*E* = constant).

All interference models assumed a log-normal error distribution for per capita consumption and were fitted by maximum likelihood. Residual scale was estimated for every model and counted as a fitted parameter when calculating the Akaike information criterion corrected for small samples (AICc). The model with the lowest AICc was preferred, while models within 2 units were considered similarly supported [31]. Larger differences indicated progressively weaker support rather than a significance threshold. Parameter uncertainty was quantified with percentile 95% confidence intervals from 999 nonparametric bootstrap resamples of arenas within predator density.

The intensity of scramble competition (*I*) at each predator density was calculated as:

(5) $$I_{P}=\frac{E_{1}-E_{P}}{E_{1}}$$

Here, *E*_1_ is per capita consumption at *P* = 1, and *E*_P_ is per capita consumption at predator density *P*.

Interference models and bootstrap analyses were implemented in R using the nlstools package v.2.1-0.

### 2.4. Predatory Behavior

The predatory behavior of adult-female *A. custos* was observed separately against second- and fourth-instar *T. absoluta* larvae. Twenty larvae of a single instar were placed on tomato leaflets in an experimental container, after which one adult female that had been deprived of prey for 24 h was introduced.

Two observers alternated to continuously monitor each replicate under a stereomicroscope (Nikon Eclipse Ts2; Nikon Corporation, Tokyo, Japan) for a total of 12 h. Searching time was defined as the interval between predator introduction and the first physical contact with a prey individual. Piercing time was defined as the interval between first contact and successful stylet insertion. The sequence of prey examination, probing, stylet insertion, and feeding was recorded, together with the body region at which the stylet was inserted.

Each prey-instar treatment was replicated 15 times, and each predator was used only once.

### 2.5. Greenhouse Cage Trial of Predation Performance Under Cage Conditions

The capacity of adult-female *A. custos* to suppress *T. absoluta* populations and limit the accumulation of plant damage was evaluated under greenhouse cage trials. Here, greenhouse cage trial refers to intact potted plants held in cages under greenhouse conditions. This setting increased spatial and structural complexity relative to laboratory arenas while preventing the immigration and emigration of both *A. custos* and *T. absoluta*. Greenhouse environmental conditions were maintained as described in Section 2.1.

Three tomato plants of a similar size and developmental stage were placed in each mesh cage (50 × 50 × 50 cm). Five second-instar *T. absoluta* larvae (24 h after molt) were introduced onto each plant, resulting in an initial density of 15 larvae per cage. Larvae were allowed to establish on the plants for 24 h before predators were released. Treatment cages received three adult-female *A. custos* (7 d old), whereas control cages received no predators. Each treatment was replicated in 3 independent cages arranged in a randomized block design. Cages, rather than individual plants or leaves, were treated as the experimental units. The number of surviving *T. absoluta* larvae and the cumulative number of damaged leaves were recorded daily after predator release over a 15-day period. At each assessment, all leaves were visually inspected. A leaf was classified as damaged when at least one visible *T. absoluta* feeding mine was present. Each damaged leaf was counted only once, and newly damaged leaves identified at subsequent assessments were added to the cumulative total.

Leaf-damage suppression was calculated as:

(6) $$Leaf-damage\;suppression\;(\%)=\frac{\left(X_{ck}-X_{pred}\right)}{X_{ck}}\times 100$$

where *X_CK_* is the mean leaf damage in the control group and *X_pred_* is the mean leaf damage in the predator treatment group.

Corrected larval control efficacy was calculated as:

(7) $$Corrected\;control\;efficacy\;(\%)=\frac{\left(S_{ck}-S_{pred}\right)}{S_{ck}}\times 100$$

Here, *S_ck_* and *S_pred_* are the mean larval survival rates in control and predator-release cages, respectively. For each cage, the area under the progress curve (AUPC) was calculated by trapezoidal integration across 15 daily assessments [33]. AUPC was calculated separately for detectable active larvae (larva-days) and cumulative damaged leaves (leaf-days).

### 2.6. Olfactory Bioassays with a Y-Tube Olfactometer

The olfactory responses of *A. custos* were evaluated using a glass Y-tube olfactometer (Model XL-YT120-20; Nanjing Xuelai Biotechnology Co., Ltd., Nanjing, China) consisting of a main arm 150 mm long, 20 mm internal diameter and two side arms 150 mm long, 20 mm internal diameter with an internal angle of 75° between the two side arms. Charcoal-filtered and humidified air was delivered through each arm at a rate of 150 mL min^−1^ using a positive/negative pressure oil-free vacuum pump (220 V, mean flow rate 25 L/min, relative vacuum −88 kPa, adjustable; Nanjing Xuelai Biotechnology Co., Ltd., Nanjing, China). Bioassays were conducted under the controlled environmental conditions described in Section 2.1, with diffuse laboratory light (i.e., without direct sunlight). Four odor-source comparisons were tested: (1) *T. absoluta*-infested tomato plants versus healthy tomato plants, tested with adult females; (2) previously infested tomato plants from which all larvae had been removed versus healthy tomato plants, tested with adult females; (3) *T. absoluta*-infested tomato plants versus healthy tomato plants, tested with adult males; and (4) *T. absoluta* larvae versus clean air, tested with adult females.

Infested plants were exposed to 50 second-instar *T. absoluta* larvae for 36 h. For previously infested plants, all larvae were then removed while damaged tissue remained as the odor source. Ten plants were prepared for each plant treatment. Adults within seven days after emergence were deprived of prey for 12 h before testing. One predator was introduced into the main arm and observed for up to 10 min. A valid choice occurred when the insect crossed a side-arm midpoint and remained there for at least 10 s. Insects making no valid choice were classified as non-respondents. Each odor comparison included five blocks of 15 insects (75 insects total), and each predator was tested once. Odor positions were alternated, and the Y-tube was rotated 180° after every five insects. After each block, all glassware was soaked in absolute ethanol, rinsed with distilled water, and air-dried.

### 2.7. Statistical Analysis

Raw predation, behavioral, olfactory, and greenhouse cage trial data were organized with Microsoft Excel 2019 (Microsoft Corp., Redmond, WA, USA). Statistical analyses were conducted in R. Functional-response models used frair; interference likelihoods and bootstrap resampling used stats and boot; mixed-effects models used lme4 v.2.0-6; and AUPC comparisons used stats. Figures were produced with ggplot2 v.4.0.3 and assembled with patchwork v.1.3.2. Daily prey consumption was compared among initial densities within each predator–prey combination using stats::aov followed by TukeyHSD. Greenhouse observations were analyzed with Gaussian linear mixed-effects models fitted by maximum likelihood using lme4::lmer with REML = FALSE. Treatment, categorical sampling day, and their interaction were fixed effects, and cage was included as a random intercept. The treatment-dependent time course was evaluated by a likelihood-ratio comparison of the full interaction model with the additive model. All tests were two-sided with α = 0.05, and quantitative data are reported as mean ± SE.

Functional-response form, parameter uncertainty, and model support were determined from the depletion-aware models described in Section 2.2. The AICc difference was defined as AICcflexpnr–AICcRogers II. Interference-model selection and uncertainty estimation followed Section 2.3.

Each valid Y-tube trial produced a binary arm choice. Pooled choices for each odor pair were analyzed using a chi-square goodness-of-fit test against an equal-choice expectation (50%:50%). Non-respondents were excluded. Statistical significance was defined as *p* < 0.05.

Cage-level AUPC values were compared between predator-release and control treatments using two-sided Welch two-sample *t* tests. Final-day leaf-damage suppression and corrected larval control efficacy were calculated from treatment means using Equations (6) and (7).

## 3. Results

### 3.1. Functional Responses of A. custos to T. absoluta Larvae

#### 3.1.1. Effects of Predator Stage and Sex on Predation of Fourth-Instar Larvae

**Figure 1:**
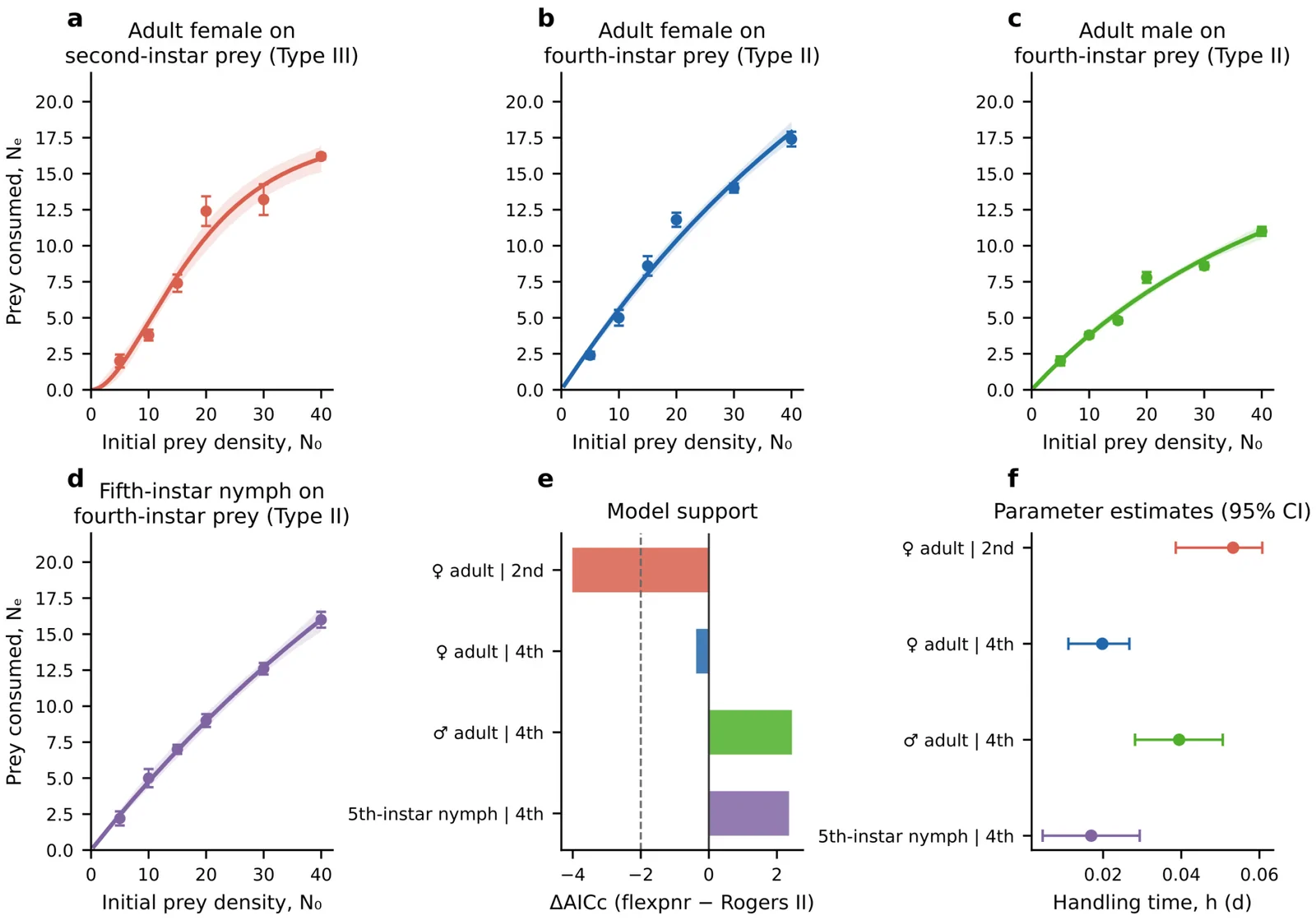
Functional responses of *Arma custos* to *Tuta absoluta* larvae under non-replacement conditions. (**a**–**d**) Observed consumption (mean ± SE, *n* = 5 arenas) and selected depletion-aware maximum-likelihood curves; shaded regions are 95% bootstrap confidence bands (999 resamples). (**e**) Difference in small-sample Akaike information criterion between flexpnr and Rogers II; values below −2 support Type III. (**f**) Handling-time estimates with 95% bootstrap CIs. Adult females on second-instar prey showed Type III support; the other combinations were retained as Type II under the prespecified ΔAICc ≥ 2 rule.

Functional-response analyses focused on larvae. Mean consumption increased with initial density in all four predator–prey combinations (Figure 1a–d). From five to 40 larvae per container, consumption increased from 2.0 ± 0.4 to 16.2 ± 0.2 for females attacking second-instar larvae. For fourth-instar larvae, female consumption increased from 2.4 ± 0.2 to 17.4 ± 0.5. Male consumption increased from 2.0 ± 0.3 to 11.0 ± 0.3, and nymphal consumption from 2.2 ± 0.5 to 16.0 ± 0.5.

Within each predator–prey combination, daily consumption differed among initial prey densities (one-way ANOVA and Tukey’s HSD, *p* < 0.0001; Table 1), supporting subsequent depletion-aware functional-response analysis. Descriptive consumption percentages are provided in Table 2.

#### 3.1.2. Effect of Prey Instar on Predation by Adult Females

Model selection supported a Type III response for adult females feeding on second-instar larvae: flexpnr was preferred to Rogers II (AICc = 123.95 versus 127.97; ΔAICc = −4.01). The fitted parameters were *b* = 0.078 (95% CI, 0.014–0.331), *q* = 1.04 (0.40–1.76), and *h* = 0.053 d (0.039–0.061; Figure 1a). For females feeding on fourth-instar larvae, flexpnr was only 0.37 AICc units lower than Rogers II (113.41 versus 113.79). Rogers II was therefore retained under the prespecified parsimony rule (Figure 1b).

Rogers II was also selected for adult males and fifth-instar nymphs feeding on fourth-instar larvae. Rogers II versus flexpnr AICc values were 104.03 versus 106.47 for males and 111.18 versus 113.54 for nymphs. For fourth-instar prey, adult females had a = 0.918 (95% CI, 0.769–1.078) and *h* = 0.020 d (0.011–0.027). Adult males had *a* = 0.563 (0.486–0.654) and *h* = 0.039 d (0.028–0.051). Fifth-instar nymphs had *a* = 0.702 (0.578–0.882) and *h* = 0.017 d (0.005–0.029; Figure 1b–d; Table 3).

#### 3.1.3. Model Support and Handling-Time Uncertainty

The AICc comparison supported flexpnr only for adult females attacking second-instar larvae; the other three combinations did not meet the prespecified threshold for retaining the more flexible model (Figure 1e). Bootstrap intervals for handling time are shown in Figure 1f. Because attack coefficients differ in definition between model forms and the assays were not designed as a single joint comparison, these estimates were not used to rank predator stages, sexes, or prey instars.

### 3.2. Intraspecific Interference Among Adult Females

**Figure 2:**
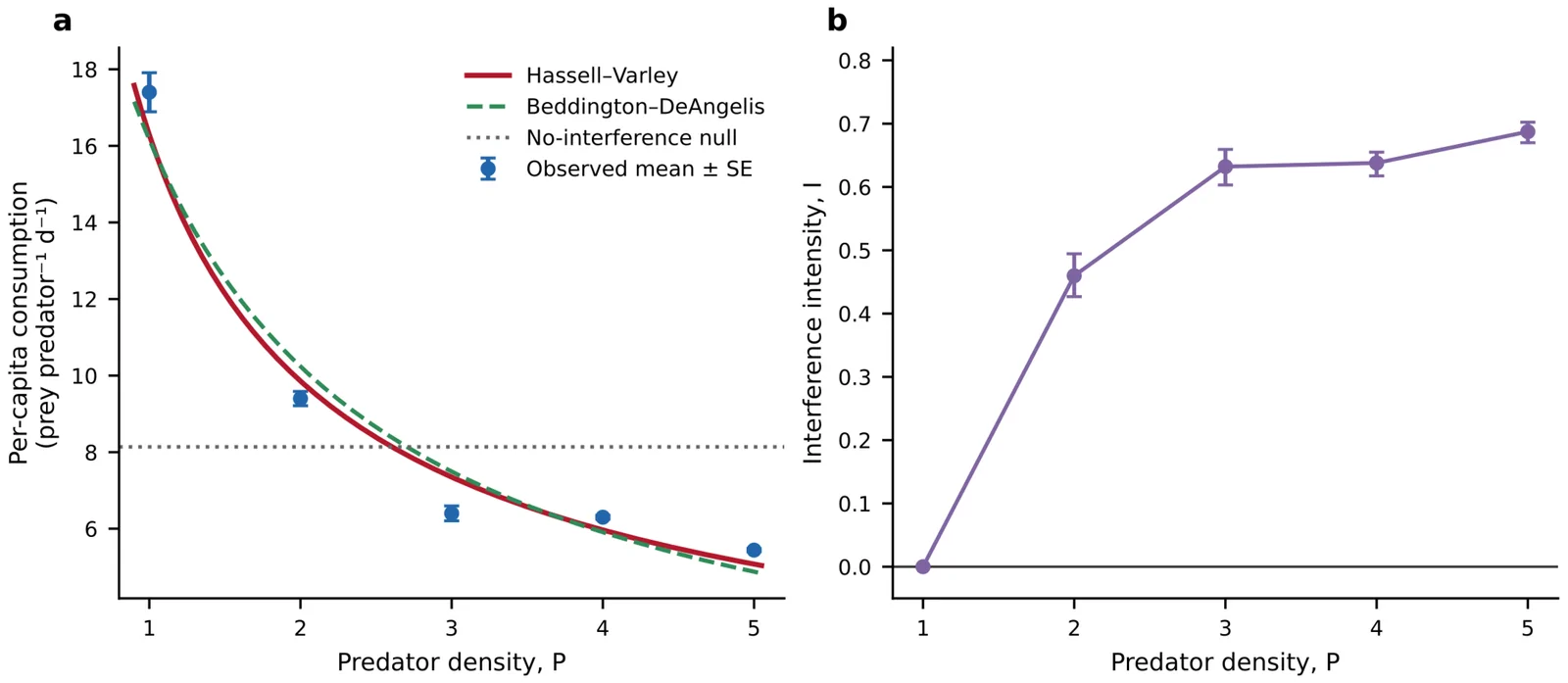
Density dependence in prey consumption by adult-female *Arma custos*. Forty fourth-instar *Tuta absoluta* larvae were offered for 24 h at predator densities *P* = 1–5 (*n* = 5 arenas per density). (**a**) Per capita consumption (mean ± SE) with Hassell–Varley (solid), Beddington–DeAngelis (dashed), and no-interference null (dotted) fits. Hassell–Varley was favored (AICc = −40.66) over Beddington–DeAngelis (AICc = −31.05; ΔAICc = 9.60) and the null (AICc = 32.38); *m* = 0.725 (95% CI 0.693–0.755). (**b**) Interference intensity relative to *P* = 1; error bars are 95% bootstrap CIs. Because prey availability per predator also declined with *P*, this confined-arena result should not be attributed solely to behavioral interference.

Total consumption increased with predator density, whereas per capita consumption declined (Figure 2a; Table 4). Per capita consumption fell from 17.40 ± 0.51 prey predator^−1^ d^−1^ at *P* = 1 to 5.44 ± 0.04 at *P* = 5. Both interference models substantially outperformed the no-interference model (AICc = 32.38). The Hassell–Varley model fitted best (AICc = −40.66), followed by the Beddington–DeAngelis model (AICc = −31.05; ΔAICc = 9.60).

The Hassell–Varley model estimated *Q* = 16.29 prey predator^−1^ d^−1^ (95% CI, 15.60–16.91) and *m* = 0.725 (0.693–0.755). Beddington–DeAngelis estimates were *E*_0_ = 16.16 prey predator^−1^ d^−1^ (15.42–16.89) and *γ* = 0.579 (0.534–0.626). Scramble-competition intensity increased from 0 at *P* = 1 to 0.460, 0.632, 0.638, and 0.687 at *P* = 2–5 (Figure 2b), indicating progressively lower per capita efficiency as predator density increased.

### 3.3. Predatory Behavior of Adult Females

**Figure 3:**
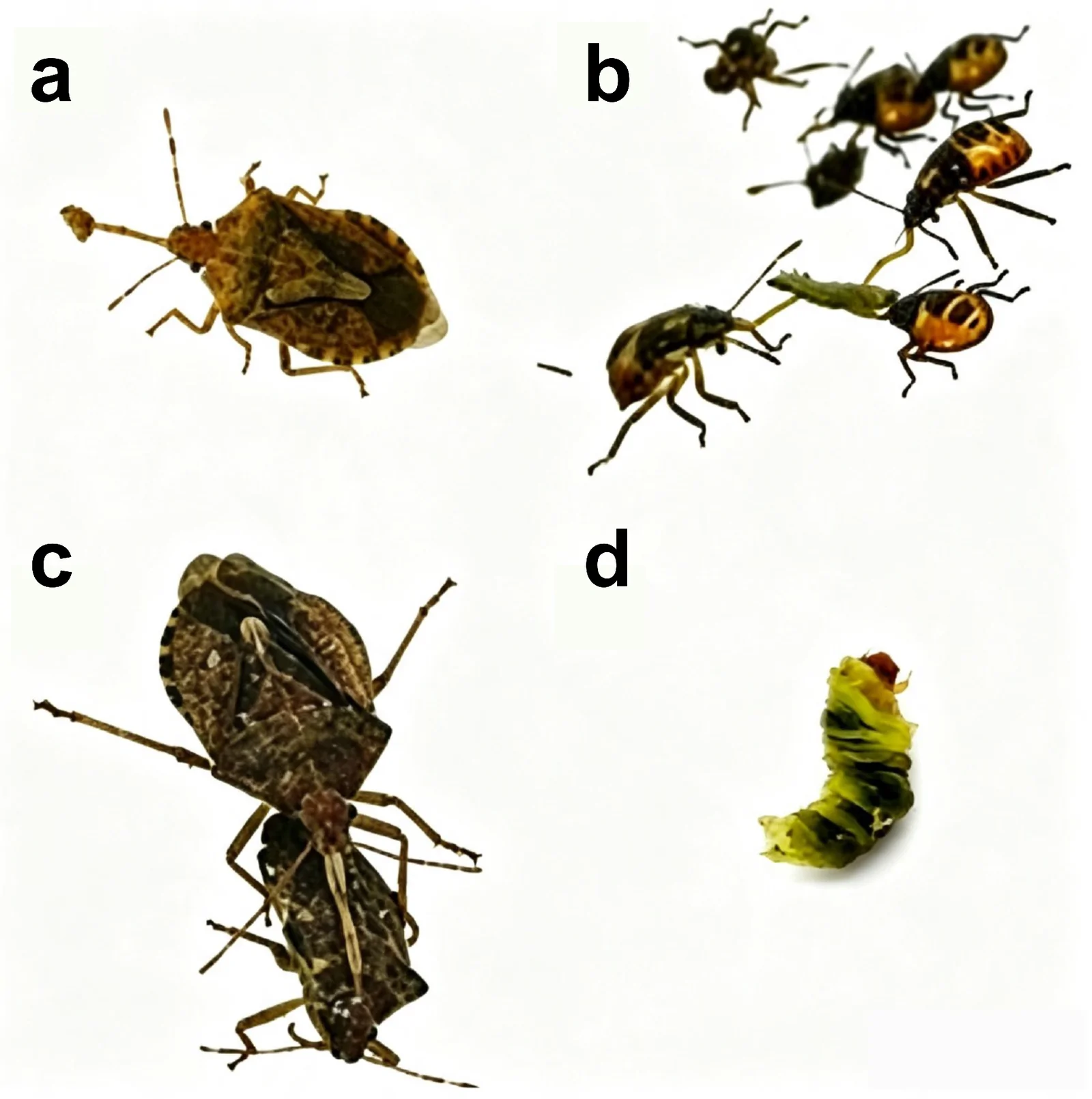
Predation behavior of *Arma custos* on *Tuta absoluta* larvae. (**a**) Female adult preying singly on a 4th instar larva; (**b**) nymphs feeding cooperatively on a 4th instar larva; (**c**) cannibalism among female adults under starvation conditions; (**d**) a 4th instar larva desiccated after predation.

Adult-female *A. custos* successfully attacked both second- and fourth-instar *T. absoluta* larvae. Predation generally consisted of four observable phases: searching, prey examination and probing, stylet insertion, and feeding. After locating a larva, females contacted it with the antennae and rostrum before stylet insertion. The stylet most often entered a soft thoracic or abdominal region (Figure 3a,d). Females occasionally lifted or partially suspended larvae while feeding. Separate photographs documented simultaneous feeding by nymphs and cannibalism among starved females (Figure 3b,c). These qualitative observations were excluded from formal behavioral comparisons.

### 3.4. Suppression of Tuta absoluta Under Greenhouse Cage Trial Conditions

**Figure 4:**
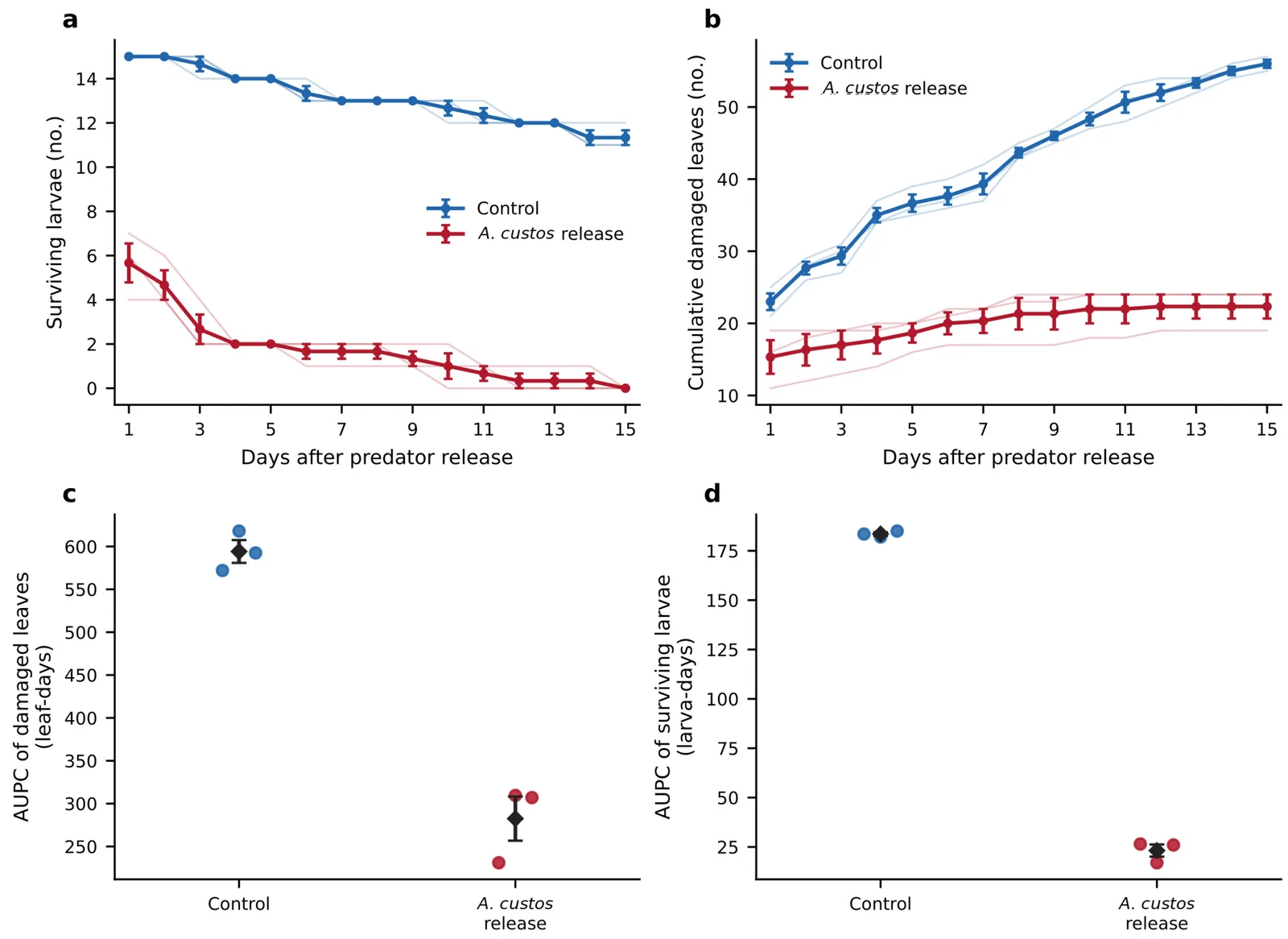
Effects of adult-female *Arma custos* release in greenhouse cage trial cages. Each cage contained three tomato plants and 15 second-instar *Tuta absoluta* larvae; release cages received three predators (*n* = 3 independent cages per treatment). (**a**,**b**) Daily surviving larvae and cumulative damaged leaves. Thin lines are individual cages; thick points and lines are treatment means ± SE. Cage-level Gaussian mixed models included a random cage intercept and categorical day, treatment and their interaction. Treatment-dependent trajectories were supported for surviving larvae (interaction likelihood-ratio χ^2^_14_ = 40.23, *p* = 0.0002) and damaged leaves (χ^2^_14_ = 335.81, *p* < 0.0001). (**c**,**d**) Cage-level area under the progress curve (AUPC; trapezoidal integration); circles are cages and diamonds are means ± SE. Welch tests gave *t* = 10.75, df = 3.00, *p* = 0.0017 for damaged leaves and *t* = 50.01, df = 2.31, *p* = 0.0001 for surviving larvae.

Predator release altered the trajectories of detectable active larvae and cumulative leaf damage during the 15-day greenhouse cage trial (Figure 4a,b). Cage-level mixed-effects models supported treatment-by-day interactions for surviving larvae (likelihood-ratio χ^2^_14_ = 40.23, *p* = 0.0002) and damaged leaves (*χ*^2^_14_ = 335.81, *p* < 0.0001). At day 15, no active larvae were detected in predator-release cages, whereas control cages retained 11.33 ± 0.33 larvae. Cumulative damaged leaves reached 56.00 ± 0.58 in controls and 22.33 ± 1.67 in predator-release cages, corresponding to 60.12% suppression. Predator release also reduced cage-level AUPC for damaged leaves from 594.17 ± 13.31 to 282.50 ± 25.76 leaf-days (Welch *t* = 10.75, df = 3.00, *p* = 0.0017; Figure 4c). Larval AUPC declined from 183.50 ± 0.87 to 23.17 ± 3.09 larva-days (Welch *t* = 50.01, df = 2.31, *p* = 0.0001; Figure 4d).

### 3.5. Olfactory Responses to Prey- and Plant-Associated Odors

**Figure 5:**
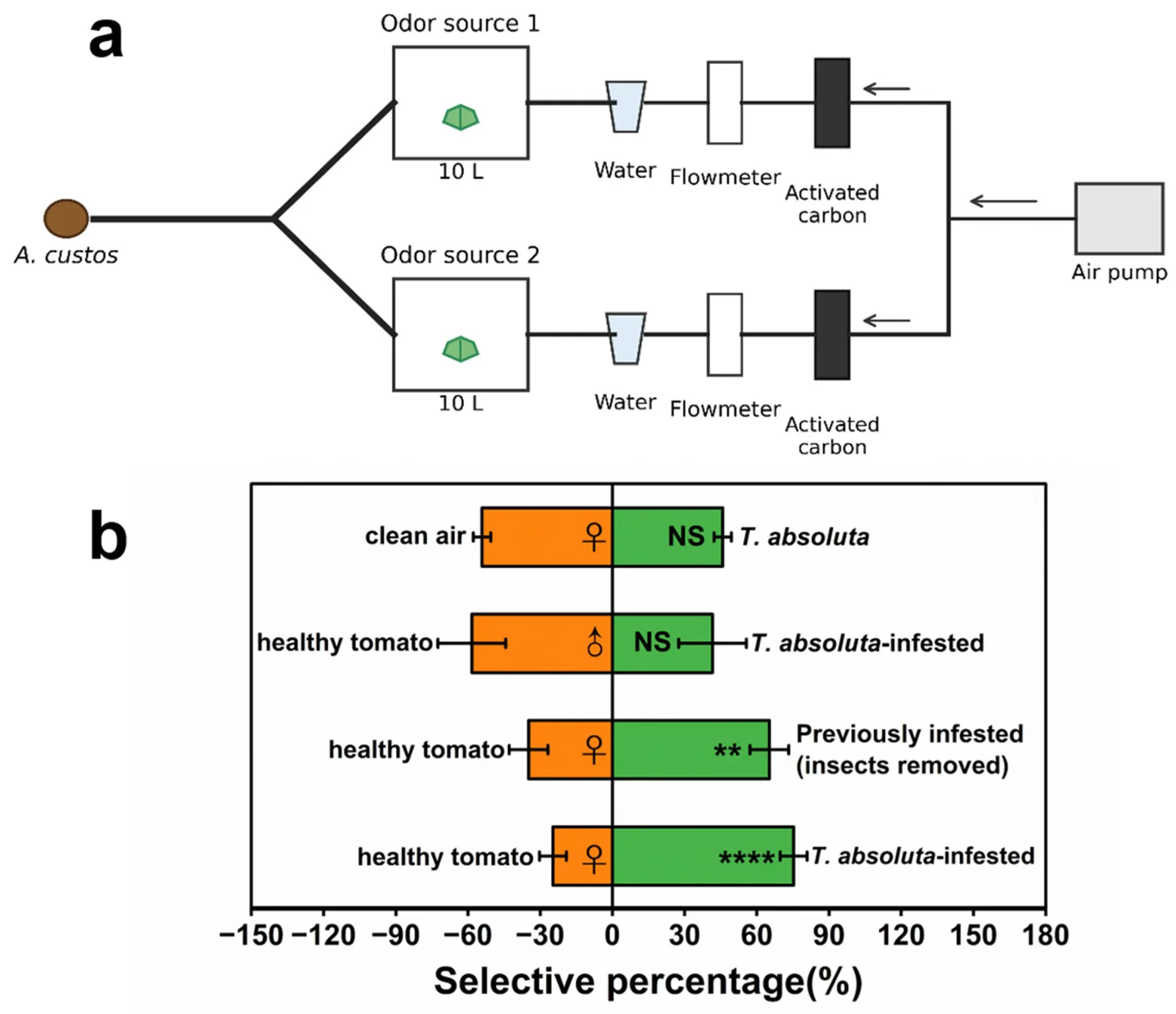
Olfactory responses of *Arma custos* in a Y-tube olfactometer. (**a**) Experimental airflow and odor-source arrangement. (**b**) Mean choice percentages ± SE across five experimental runs; left and right percentages sum to 100% within each run. Chi-square goodness-of-fit tests against 50:50 choice, calculated from the underlying individual counts, were: female, *Tuta absoluta* larvae versus clean air, *χ*^2^ = 0.053, *p* = 0.818; male, infested versus healthy tomato, χ^2^ = 0.98, *p* = 0.322; female, previously infested tomato (insects removed) versus healthy tomato, χ^2^ = 7.70, *p* = 0.0055; and female, infested versus healthy tomato, χ^2^ = 23.54, *p* < 0.0001. Asterisks indicate significant differences from the 50:50 choice expectation: ** *p* < 0.01, **** *p* < 0.0001.

Adult-female *A. custos* showed a significant preference for tomato plants infested with *T. absoluta* over healthy plants (χ^2^ = 23.54, *p* < 0.0001; Figure 5). Females also preferred previously infested plants from which larvae had been removed over undamaged plants (χ^2^ = 7.70, *p* = 0.00553; Figure 5). These responses indicate that cues retained by damaged plants remained attractive in the absence of actively feeding larvae. In contrast, adult males showed no significant preference between infested and healthy tomato plants (χ^2^ = 0.98, *p* = 0.32177; Figure 5). In the comparison between *T. absoluta* larvae and clean air, adult females did not show a significant preference for larvae (χ^2^ = 0.053, *p* = 0.81847; Figure 5).

## 4. Discussion

Our findings support the further evaluation of *A. custos* as a complementary predator of *T. absoluta* larvae by linking laboratory predation with reduced leaf damage in greenhouse cages. Its practical contribution to IPM will depend on prey accessibility, predator density, and performance under crop conditions beyond the tested cages.

**Figure A1:**
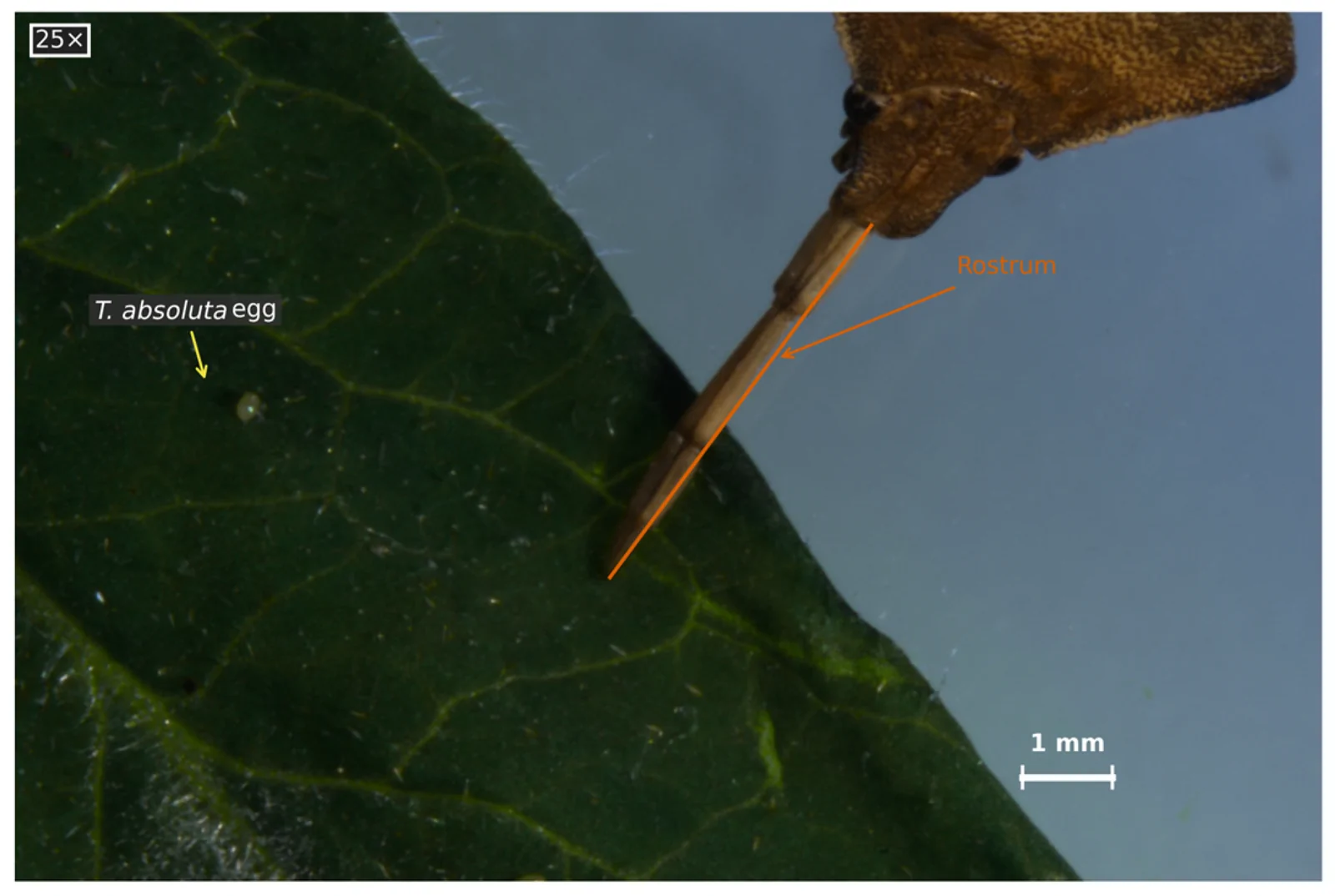
Morphological comparison illustrating the relative sizes of the rostrum of the predator *Arma custos* and the eggs of the tomato leafminer, *Tuta absoluta*.

Our functional-response results can be compared with studies that differ in both predator and prey stage. Liu et al. reported a Type II response for adults of *A. custos* offered Colorado potato beetle eggs and young larvae [34]. In contrast, Type III responses have been reported for *M. pygmaeus* and *N. tenuis* feeding on *T. absoluta* eggs [35]. Van Lenteren et al. likewise examined egg-density responses of three mirid predators [14]. Thus, the Type III support in our adult-female–second-instar treatment is not evidence of a species-wide response type: the form may depend on the prey stage, predator identity, assay design, and fitted model. We did not statistically test differences among all predator stages and sexes in a single joint model. In particular, attack coefficients from Rogers II and flexpnr are not directly comparable, and several flexpnr bootstrap intervals were wide. We therefore interpret the fitted forms within each predator–prey combination rather than ranking the four combinations by their separate parameter estimates [25,26,27,28]. Type III response form can reflect density-dependent changes in detection or attack, but our single-prey experiment cannot distinguish learning from other processes or demonstrate population-level stabilization [27,36,37]. Switching, in particular, requires an alternative prey type and was not tested here [37]. *A. custos* rarely consumed *T. absoluta* eggs in our assay but attacked both second- and fourth-instar larvae (Figure A1). This pattern differs from studies centered on egg predation by mirids [13,14,15,35] and suggests a possible complementary role across pest stages. It does not show that *A. custos* can reach larvae protected inside intact leaf mines. Our laboratory exposure and the small arenas may increase prey accessibility; direct observations on intact mined leaves are needed before larval-stage complementarity can be claimed under crop conditions.

The decline in per capita consumption under fixed total prey availability suggests diminishing returns from increasing predator density in confined arenas. Predator-dependent models captured this pattern, but the design does not isolate direct behavioral interference from reduced prey availability per predator. Predator-dependent models formalize this decline [30,31,32,38], and Michaelides et al. found non-independent interactions between pairs of mirid predators attacking *T. absoluta* eggs at high prey density [35]. Their two-predator, egg-based system is not directly comparable with our adult-female, larva-based density series. Moreover, our design changed the prey-to-predator ratio at the same time as predator density. Resource dilution, crowding, and direct behavioral interference remain confounded; m should be interpreted as a density-associated pattern in this assay, not proof of a particular mechanism or an optimal greenhouse release rate. The effect of habitat structure on predator interference reinforces the need to test several release densities on whole plants [39].

Adult females preferred odors from infested tomato plants to those from healthy plants, and preference persisted after visible larvae were removed. In other predator species, De Backer et al. observed the attraction of *M. pygmaeus* to *T. absoluta*-infested tomato plants [40], while Ayelo et al. identified attractive terpenes in herbivore-induced tomato volatiles for *N. tenuis* [41]. These studies make a plant-associated odor explanation plausible, but they do not identify the compounds responsible for *A. custos* orientation. Our odor sources could still contain frass, silk, or residual prey cues, and we did not include mechanically damaged plants or analyze headspace chemistry. Adult males showed no detectable preference in this test; that finding does not establish an inability to perceive the odors or a fixed sex difference across ages and physiological states [42]. Volatile profiling followed by synthetic-compound and electrophysiological tests would resolve the cue and its behavioral specificity.

The cage trial links laboratory predation with reduced pest abundance and cumulative leaf damage on tomato plants. These outcomes are broadly consistent with greenhouse studies in which preplant release of *N. tenuis* contributed to *T. absoluta* management [10] and releases of *M. basicornis* or the egg parasitoid *Trichogramma pretiosum* reduced pest density and plant damage [43]. Differences in natural enemy, release timing, crop setting, and outcome definition preclude a numerical efficacy comparison. The larger reduction in final active-larva counts than in cumulative leaf damage is expected because earlier mining damage remains visible. In addition, the disappearance of detectable larvae does not establish that all larvae were consumed: pupation, emergence, natural mortality, and imperfect recovery were not ruled out. Cages may also restrict dispersal. Our trial therefore supports suppression under the tested cage conditions, but not a claim of field efficacy or an optimized release program.

Taken together, the findings provide qualified support for the three tested hypotheses. The first hypothesis received partial support: response form differed between the adult-female assays on second- and fourth-instar larvae, but formal between-group differences in fitted parameters across predator stages and sexes were not established. The second hypothesis was supported descriptively: per capita consumption declined as predator density rose under fixed total prey supply, although direct interference was not isolated from declining prey availability per predator. The third hypothesis was partly supported: females, but not males, preferred infested-plant odors in the tested contrasts, and predator release reduced detectable larvae and cumulative leaf damage in cages. These findings justify further evaluation of *A. custos* as a complementary predator of larval *T. absoluta.* Priority experiments should measure access to larvae within intact mines, separate predator density from prey-to-predator ratio, identify attractive odor cues, and compare release timing and density in larger greenhouse and field trials.

## Figures and Tables

**Table 1:** Daily predation number of *Arma custos* on *Tuta absoluta* larvae at different prey densities. Values are mean ± SE (*n* = 5 independent arenas). Within each predator–prey combination (row), means followed by the same lowercase letter are not significantly different according to Tukey’s HSD following one-way ANOVA (*p* < 0.05).

*A. custos* Stage	*T. absoluta* Instar	5 Prey	10 Prey	15 Prey	20 Prey	30 Prey	40 Prey
Fifth-instar nymph	fourth	2.2 ± 0.5 a	5.0 ± 0.6 b	7.0 ± 0.3 bc	9.0 ± 0.4 c	12.6 ± 0.4 d	16.0 ± 0.5 e
Adult female	fourth	2.4 ± 0.2 a	5.0 ± 0.5 b	8.6 ± 0.7 c	11.8 ± 0.5 d	14.0 ± 0.3 e	17.4 ± 0.5 f
Adult male	fourth	2.0 ± 0.3 a	3.8 ± 0.2 b	4.8 ± 0.2 b	7.8 ± 0.4 c	8.6 ± 0.2 c	11.0 ± 0.3 d
Adult female	second	2.0 ± 0.4 a	3.8 ± 0.4 a	7.4 ± 0.6 b	12.4 ± 1.0 c	13.2 ± 1.1 cd	16.2 ± 0.2 d

**Table 2:** Daily predation rate (%) of *Arma custos* on *Tuta absoluta* larvae at different prey densities. Predation rate = (number of prey consumed/initial number of prey) × 100%. Within each predator–prey combination (row), means followed by the same lowercase letter are not significantly different according to Tukey’s HSD following one-way ANOVA (*p* < 0.05).

*A. custos* Stage	*T. absoluta* Instar	5 Prey	10 Prey	15 Prey	20 Prey	30 Prey	40 Prey
Fifth-instar nymph	fourth	44.0 a	50.0 b	46.7 bc	45.0 c	42.0 d	40.0 e
Adult female	fourth	48.0 a	50.0 b	57.3 c	59.0 d	46.7 e	43.5 f
Adult male	fourth	40.0 a	38.0 b	32.0 b	39.0 c	28.7 c	27.5 d
Adult female	second	40.0 a	38.0 a	49.3 b	62.0 c	44.0 cd	40.5 d

**Table 3:** Depletion-aware functional-response fits for *Arma custos* preying on *Tuta absoluta* larvae.

Stage	Prey Instar	Selected Model	*a* or *b* (95% CI)	*q* (95% CI)	*h*, d (95% CI)	AICc II	1/h (Prey Predator^−1^ d^−1^)	AICc Flex
Female adult	2nd	flexpnr (Type III)	*b* = 0.078 (0.014–0.331)	1.04 (0.40–1.76)	0.053 (0.039–0.061)	127.97	18.87	123.95
Female adult	4th	Rogers II (Type II)	*a* = 0.918 (0.769–1.078)	—	0.020 (0.011–0.027)	113.79	50.00	113.41
Male adult	4th	Rogers II (Type II)	*a* = 0.563 (0.486–0.654)	—	0.039 (0.028–0.051)	104.03	25.64	106.47
5th nymph	4th	Rogers II (Type II)	*a* = 0.702 (0.578–0.882)	—	0.017 (0.005–0.029)	111.18	58.82	113.54

Models were fitted by binomial maximum likelihood under non-replacement conditions using the Lambert-W form of Rogers’ random-predator equation. The flexible model uses *a* = *bN*_0_*^q^*; *q* > 0 supports a sigmoidal response. flexpnr was selected only when its AICc was at least 2 units lower than Rogers II. Intervals are percentile 95% CIs from 999 arena-level bootstrap resamples. *T* = 1 d.

**Table 4:** Density-dependent intraspecific interference among adult-female *Arma custos*.

Predator Density, P	Total Consumed	Per Capita Consumption, E	Interference Intensity, I
1	17.4 ± 0.5 a	17.40 ± 0.51	0.000
2	18.8 ± 0.4 ab	9.40 ± 0.19	0.460
3	19.2 ± 0.6 b	6.40 ± 0.19	0.632
4	25.2 ± 0.2 c	6.30 ± 0.05	0.638
5	27.2 ± 0.2 d	5.44 ± 0.04	0.687

Initial prey density was 40 fourth-instar larvae per arena; values are mean ± SE (*n* = 5 arenas). Within each row, means followed by the same lowercase letter are not significantly different based on Tukey’s HSD after one-way ANOVA (F_4_,_20_ = 115.17, *p* < 0.0001). Log-normal maximum-likelihood fits (999 bootstrap resamples) favored Hassell–Varley: *Q* = 16.29 (15.60–16.91), *m* = 0.725 (0.693–0.755), AICc = −40.66; Beddington–DeAngelis: *E*_0_ = 16.16 (15.42–16.89), *γ* = 0.579 (0.534–0.626), AICc = −31.05; and null AICc = 32.38. Residual scale was counted as a fitted parameter in every AICc.

## Data Availability

The original contributions presented in this study are included in the Appendix A. Further inquiries can be directed to the corresponding authors.

## Abbreviations

The following abbreviations are used in this manuscript:

RH	Relative Humidity
SE	Standard Error
ANOVA	Analysis of Variance
χ^2^	Chi-square
df	Degrees of freedom
AUPC	Area under the progress curve
IPM	Integrated Pest Management
